# Accounting for the gross ecosystem product (GEP) of forests in nature reserves—taking the Taishan Scenic and Historic Spot as an example

**DOI:** 10.1371/journal.pone.0320075

**Published:** 2025-03-25

**Authors:** Chongqing Xu, Teng Zhao, Yuting Shao, Xiaoxia Li, Guihuan Yan

**Affiliations:** 1 Qilu University of Technology, Jinan, Shandong, China,; 2 Ecology Institute of Shandong Academy of Sciences (China-Japan Friendship Biotechnology Research Center of Shandong Academy of Sciences), Jinan, Shandong, China,; 3 Shandong Technology Innovation Center of Carbon Neutrality, Jinan, Shandong, China; Tennessee State University, UNITED STATES OF AMERICA

## Abstract

How to measure the economic value of forest ecosystems is an important research topic for sustainable development. Existing quantitative systems for ecosystem values were compared and analyzed on the basis of characteristics of forest ecosystems in the Taishan Scenic and Historic Spot with reference to the Technical Guidelines for Accounting for the Gross Value of Ecological Products in Shandong Province. Two new indicators, namely, forest protection and scientific research and education, were added to the accounting system, forming a “forest sample” applicable to the accounting of GEP in nature reserves to better understand the synergistic effect between conservation and development. The forest GEP of the Taishan Scenic and Historic Spot in 2022 and 2023 was calculated according to the index system developed in this work, and the results of these calculations were used to analyze the environmental changes and current status of the Taishan forest ecosystems. The results revealed that the GEP of forest ecological products in Taishan Scenic and Historic Spot in 2023 was 4.478 billion yuan, the value of the unit area was 496,900 yuan per hectare, and the value of the regulating service was 2.312 billion yuan, which was slightly greater than the value of the cultural service, at 2.186 billion yuan. The values of secondary classification of forest ecosystems were determined to be in the following order: evergreen coniferous forests>  deciduous broad-leaved forests>  mixed coniferous and broad-leaved forests>  sparse forests. Compared with that in 2022, the GEP value of forests in Taishan Scenic and Historic Spot has increased by 110%, and ecological protection is more effective.

## 1 Introduction

Research related to the system of accounting for the value of ecosystem services has been an increasingly popular topic in the field of sustainable development. Costanza et al. defined ecosystem services as all the benefits that humans derive from nature[1] and valued 17 ecosystem services[2]. The Millennium Ecosystem Assessment (MA) further standardized the classification of ecosystem services into four categories, namely, provisioning, regulating, supporting and cultural services[3], which provided the basis for the quantification of ecosystem services. The system of environmental‒economic accounting—ecosystem accounting[4]—provides a detailed description of how to quantify ecosystem services and was adopted by the UN Statistical Commission in 2021[5]. Studies have shown that scientific quantification of the value of ecosystem services promotes the efficient use of natural resources and can provide a basis for improved policies[6–8]. The concept of ecological products proposed by China is based on the concept of ecosystem service functions. This term refers to the contribution of goods and services provided by ecosystems to economic and other human activities[9]. The concept most similar to that of ecological products is that of ecosystem services[10]. However, ecological products encompass not only natural elements but also the positive benefits of ecosystems for human well-being[11]. The GEP translates ecosystem services into a common monetary form that is easy to understand and can highlight the value of nature and the contribution of ecosystems to human well-being[12]. In recent years, China has issued a steady stream of national and local standards for accounting for GEP[9,13,14]. A comparison of the studies revealed that the forest ecological product indicators in the different standards cover different scopes. Harmonization of the scope of accounting is therefore extremely important.

As the mainstay of terrestrial ecosystems, the services of forest ecosystems bring enormous ecological wealth to society, so it is important to assess the multiple values associated with these ecosystems. In recent years, relevant research accounting for the value of forest ecological products has improved. In 2022, the state promulgated norms for accounting for gross ecological products[9] that cover the service functions of six major ecosystems, namely, forests, grasslands, wetlands, farmlands, deserts, and oceans, and form an important basis for accounting for the value of ecological products with respect to administrative regional units. However, there are no uniform standards for accounting for the value of ecological products for specific geographical units. Nature reserves are natural concentration areas for the protection of rare and endangered wild species, as well as key conservation areas for the ecological environment[15]. As of 2009, there were a total of 2,538 nature reserves in China[16]. Therefore, accounting for GEP in nature reserves is crucial.

Material supply, water conservation, soil conservation, carbon sequestration and nutrient release, climate regulation, air purification, and recreation and tourism are commonly associated with forest ecological products[17,18]. However, the scientific value of ecosystems to society is equally important[19]. Ecosystems can provide opportunities for the investigation and discovery of new knowledge. In nature reserves, the value of scientific research is even more important. In addition, the role of forests in the conservation of species should not be underestimated. The uniqueness and richness of forest ecological environments contribute significantly to the diversity of birds in these regions[20]. Moreover, trees and even dead wood in forest reserves fulfill the functions of forest conservation[21]. Even trees that are retained after forest harvesting can increase forest biodiversity; for example, dead retained trees are valuable substrates for the conservation of bryophytes and lichens[22]. Therefore, we infer that forest ecosystems have important species diversity conservation value as well as scientific research and education value. The two main indicators of forest conservation and scientific research and education should be added to the accounting system for forest ecological products.

Different scholars have different opinions on the categorization of the value of ecosystem services. Some scholars believe that ecosystem services should be divided into three categories: provisioning services, regulating services and cultural services[23–25]. Some scholars have classified ecosystem service categories into four categories: provisioning services, supporting services, regulating services and cultural services[26]. Current research generally assumes that supporting services are the basis for generating other services and that there may be double counting if they are involved in accounting[27]. Moreover, support services are a proxy indicator for certain intermediate services that are difficult to quantify[28]. In this work, to emphasize the value of ecosystems in providing habitats for organisms, we exclude some of the values of the biodiversity content that are duplicated by other indicators[29]. Therefore, in this work, the forest conservation biodiversity indicator is defined as the final service, i.e., the regulating service. This work does not address supply services in addition to support services. This is because mining and production activities are not allowed within the boundaries of the forest nature reserve, so there are no outputs of material supply.

Differences in accounting categories depend on differences in the basis of classification, and differences in the scope of accounting are an important reason for the low comparability of the values of ecological products. The existing accounting system is not well targeted to specific areas of nature reserves. To address these difficulties in quantifying, mortgaging, trading and realizing ecological products in nature reserves, a GEP accounting system applicable to forests in nature reserves has been developed. By accounting for the GEP in the Taishan Scenic and Historic Spot, we provide a reference for relevant decision makers and promote the sustainable development of the ecosystem.

## 2 Methods

### 2.1 Overview of the study area

**Fig. 1:**
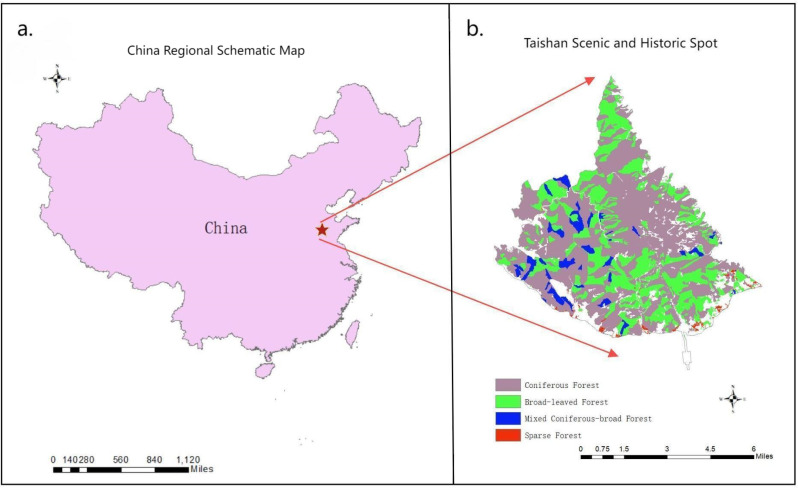
Schematic of the study area. Map of China from http://www.naturalearthdata.com/. The image is similar but not identical to the original map, so it is for reference only.

In this work, the Taishan Scenic and Historic Spot, which is a national nature reserve and a world natural and cultural double heritage location, was selected as the research object[30,31]. The Taishan Scenic and Historic Spot is located in the central part of Shandong Province and has a northern temperate continental semihumid monsoon climate. Taishan has a complex topography and a wide variety of plants, which belong to the flora of North China. The vegetation is classified as constituting a warm-temperate deciduous broad-leaved forest zone, and the vegetation coverage rate is approximately 90%. According to the tree species structure of arbor forests given in the results of the Ninth National Forest Resources Inventory of Shandong Province, the Taishan Scenic and Historic Spot has an area of 6,550.34 hectares of broad-leaved forests, 2,103.54 hectares of coniferous forests, and 358.70 hectares of mixed coniferous and broad-leaved forests. Taishan culture is nurtured and grown in the forest, bringing spiritual enjoyment, inspiration and many other intangible benefits. After years of protection and development, the Taishan Scenic and Historic Spot has become rich in forest resources, favorable environments, and ecological beauty, and it has great ecological value. Therefore, this work accounts for the GEP of forests of the Taishan Scenic and Historic Spot, with an area of 9012.58 hectares. The study area is shown in Fig 1.

### 2.2 Source of data

The data sources for this study are listed in Table 1. Data preprocessing was carried out on the following raw data to increase parameter localization. The processing included extracting meteorological data via an ArcGIS software mask, interpolating rainfall around the Taishan Scenic and Historic Spot, and screening and interpolating the number of hot weather days.

**Table 1:** Original parameter sources.

Parameter		Numerical value	Unit	Source
Engineering cost of reservoir unit capacity		74.39	yuan/m^3^	Average of actual project costs of three major reservoirs in Shandong Province: Taiping Reservoir, Guanlu Reservoir and Shuanghou Reservoir
Annual operating cost of reservoir unit capacity		0.33	yuan/(m^3^·a)	Taishan District 2023 Reservoir Flood Control Annual Repair Project
Annual depreciation rate for reservoirs		2.65%	(dimensionless)	Technical Guidelines for Accounting for the Gross Value of Ecological Products in Shandong Province
Sediment deposition coefficient		24%	(dimensionless)	
Cost of reservoir unit dredging works		31,62	yuan/m^3^	
Soil capacity weight		1.32	(t/m^3^)	
Pure content of pollutants of class i in soil	organic matter	1.77%	(dimensionless)	
	N	0.11%	(dimensionless)	
	P	0.06%	(dimensionless)	
	K	1.33%	(dimensionless)	
Unit treatment cost of pollutants in category *i*	organic matter	3649	yuan/t	
	N	3832	yuan/t	
	P	3832	yuan/t	
	K	4134	yuan/t	
Thickness of sand-covered soil		0-100	cm	
Unit cost of sand control project or unit cost of vegetation restoration		500	yuan/m^2^	
Unit treatment cost of air pollutants	SO_2_	6315.79	yuan/t	
	N_x_O	6315.79	yuan/t	
	Dust	300	yuan/t	
Carbon dioxide price		960	yuan/t·CO_2_	
Price of industrial oxygen production		850	yuan/t·O_2_	
Local living consumption electricity price		0.55	yuan/kW·h	
Value of species conservation per unit area		13350	yuan/(hm^2^·a)	Refer to Nature Reserve Species Conservation Unit values[32].
Parameters related to cultural services		nonpublic	_	Sectors related to tourism
Average wage of tourists		156	yuan/(person·day)	Income and Consumer Expenditure of the Population in 2023, published by China National Bureau of Statistics (NBS):https://www.stats.gov.cn/sj/zxfb/202401/t20240116_1946622.html
Tai’an Research and Experimental Development Fund		0.86	billion yuan/a	Department of Science and Technology of Shandong Province released the 2023 Shandong Province Science and Technology Investment Statistics Bulletin: http://kjt.shandong.gov.cn/art/2024/10/28/art_103598_10316501.html
Data on temperature, precipitation, vegetation distribution, etc.		nonpublic	_	Sectors related to tourism
DEM		_	_	Geospatial data cloud: **http://www.gscloud.cn/**
Potential evapotranspiration data		_	_	The Qinghai‒Tibet Plateau Scientific Data Center: **https://data.tpdc.ac.cn/zh-hans/data**

### 2.3 System of accounting for GEP indicators

This study refers to the Technical Guidelines for Accounting for the Gross Value of Ecological Products in Shandong Province. Innovatively, forest protection and scientific research and education were included in the GEP index system. There are no material supply outputs from forested nature reserves, and exploitative and productive activities are not carried out within their boundaries. The GEP of forest ecosystems has only two major service values: regulating services and cultural services. In this study, on the basis of the characteristics of forest ecosystem service functions, we established a forest GEP accounting index system for nature reserves, including 2 first-level indicators and 11 second-level indicators, as shown in Table 2. The specific accounting model is described in the supporting information.

**Table 2:** Indicator System for GEP Accounting of Forests in Nature Reserves.

Indicators		Methods of Accounting		Implications of the Indicators
Primary Indicators	Secondary Indicators	Physical Quantity	Value Quantity	
Regulating Service	Water Conservation	Water Balance Model[33]	Alternative Costing Method[34]	Vegetation and soil cover on forest ecosystems provide services that intercept and store the retained portion of precipitation[35].
	Soil Conservation	Modification of the Generalized Soil Loss Equation[36]		The service function of forest ecosystems protects surface soils and the organic matter contained therein and reduces the amount of soil loss.
	Climate Regulation	Vaporization Model[37]		The service function of forest ecosystems regulates environmental temperature and humidity by consuming energy through transpiration and evaporation.
	Flood Storage	Water Balance Model[33]		Vegetation in forest ecosystems regulates stormwater runoff and reduces flood peaks to achieve the service function of flood hazard reduction.
	Air Purification	Pollutant Purification Model[37]		Forest ecosystems reduce atmospheric pollutants and improve air quality through photosynthesis absorption and physical blocking.
	Carbon Sequestration	Carbon Sequestration Rate Model[37]		The service function of forest ecosystems absorbs carbon dioxide through photosynthesis and other means and fixes carbon in plants and soil.
	Oxygen Release	Oxygen Release Mechanism Model for Carbon Sequestration[36]	Market Value Approach[38]	The service function of forest ecosystems increases the oxygen content of the air by releasing oxygen through photosynthesis.
	Wind Protection and Sand Fixation	Modified Wind Erosion Modeling[39]	Recovery Cost Approach[40]	Forest ecosystems enhance soil resistance to wind through their structure to reduce the service function of wind erosion and wind-sand hazards.
	Forest Conservation Biodiversity	Statistical Surveys Act[40]	Conservation Value Method[40]	Service function of forest ecosystems conserves biological species by providing habitat for them within the boundaries of nature reserves[41].
Cultural Service	Tourism and Recreation	Statistical Surveys Act[40]	Travel Costs Act[42]	Forest ecosystems provide humans with places to relax and recuperate, bringing nonmaterial benefits to the spirit and mind.
	Scientific Research and Education	—	Results-Based Approach[43]	Plots in forest ecosystems with scientific research and educational value provide services that add to the stock of knowledge.

Ecosystem services have various functions. Regulating services are benefits derived from ecosystem regulation that can improve the human living environment, including climate regulation, water purification, water conservation, carbon sequestration and nutrient release[44]. Since most regulating service products cannot be traded directly in the market, the quantification of monetary value is difficult. To reduce the influence of subjective factors in the pricing of regulating service products, this study accounts for the value of regulating service products with reference to the cost and market value of the corresponding services[45]. The alternative cost, market value, restoration cost, and conservation value methods are utilized in regulating service accounting.

In recent years, the number of studies on cultural services has increased over time. As important ecosystem services, cultural services play an important role in promoting human well-being and social development[46]. They cover different fields, such as landscape aesthetics, inspiration, ecotourism, and recreation[47,48]. On the basis of the characteristics of forest ecosystems in nature reserves, we established a system of accounting methods for cultural services, applying the travel cost method and the outcome reference method.

The accounting of tourism and recreation indicators varies greatly across different regions. To ensure the accuracy of the accounting results, the total number of people traveling to the scenic spots was determined by counting the annual attendance of the scenic spots from the local cultural and tourism bureaus. The travel cost method is applied to account for the value of tourism and recreation services. The valuation amount includes two major elements: the time cost of tourists traveling and tourism consumption. Travel consumption includes spending on food and lodging in the scenic area, the cost of scenic area tickets, and related extended spending, such as shopping and entertainment, driven by visiting the natural scenic area.

The indicator of research and education is considered the amount of new knowledge that can be provided to humankind through plots of land with scientific research and educational value. Owing to the intangible nature of scientific research and education, it is not explored in physical terms but only in terms of its value. The valuation amount of scientific research and education is reflected in the strength of scientific research and education funding. Therefore, the value of research and education is calculated via the results-based approach, which refers to the data on the research and experimental development inputs of research and educational institutions.

## 3 Results

**Fig. 2:**
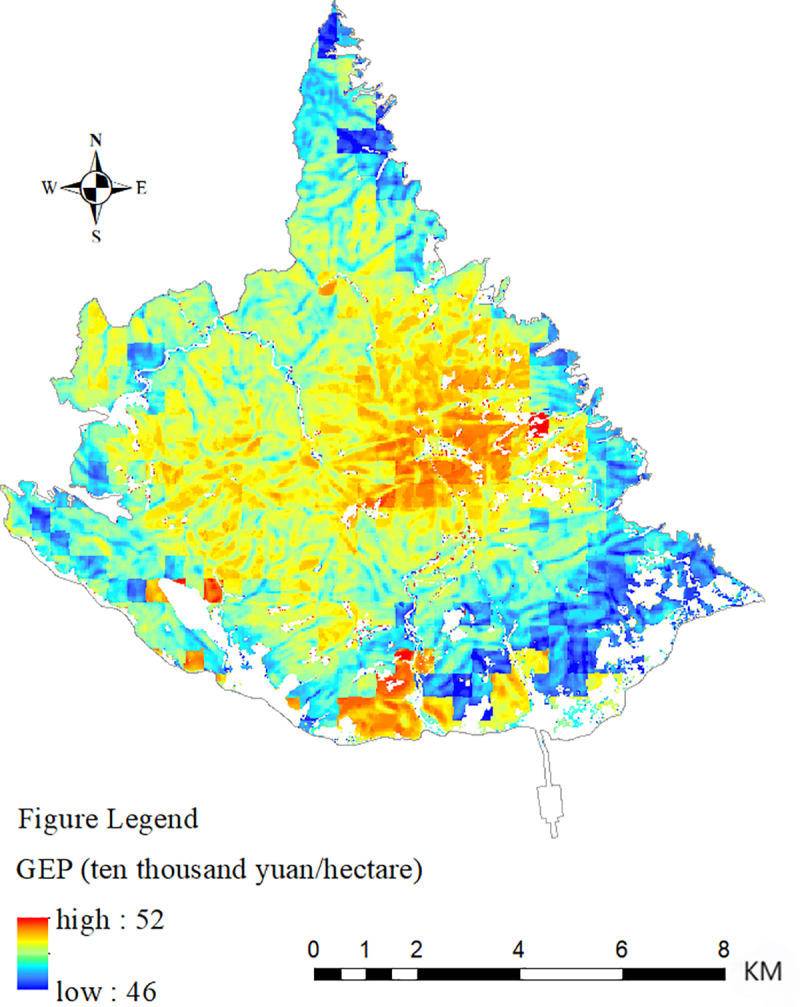
Spatial distribution of forest GEP in the Taishan Scenic and Historic Spot.

The indicator system and accounting method established above was used to account for the forest GEP of the Taishan Scenic and Historic Spot; the results are shown in Fig 2 and Table 3. In 2023, the GEP of the forest ecosystem in the Taishan Scenic and Historic Spot was 4.478 billion yuan, and the value of the unit area was 496898.54 yuan/ha. The value of regulating services, at 51.19%, was greater than that of cultural services, at 48.81%.

**Table 3:** Forest ecosystem GEP in the Taishan Scenic and Historic Spot (ten thousand yuan), 2023.

		Coniferous Forest	Broad-Leaved Forest	Mixed Coniferous–Broad-Leaved Forest	Sparse Forest	Forest Ecosystem
Regulating Service	Climate Regulation	112517.66	60913.51	9111.66	1507.61	184050.45
	Forest Conservation Biodiversity	7351.27	3982.17	593.87	104.32	12031.63
	Water Conservation	6941.89	3421.94	575.12	87.37	11026.33
	Flood Storage	5659.48	2932.16	488.31	59.92	9139.88
	Soil Conservation	3379.98	1690.77	239.01	17.25	5327.01
	Carbon Sequestration	2663.24	1442.67	215.15	37.79	4358.86
	Oxygen Release	1714.97	928.99	138.54	24.34	2806.84
	Air Purification	331.04	115.48	23.45	3.14	473.11
	Wind Protection and Sand Fixation	27.50	13.03	2.10	0.62	43.24
Cultural Service	Tourism and Recreation	132937.52	72012.07	10739.28	1886.55	217575.43
	Scientific Research and Education	611.61	331.31	49.41	8.68	1001.01
	Aggregate Value	274136.16	147784.12	22175.91	3737.60	447833.78

The total value of regulating service products in the Taishan Scenic and Historic Spot in 2023 was 2.29 billion yuan. The accounting results of the regulating service products of the Taishan Scenic and Historic Spot, in descending order, were as follows: climate regulation (1840.50 million yuan), forest conservation (120.32 million yuan), water conservation (110.26 million yuan), flood storage (91.40 million yuan), soil conservation (53.27 million yuan), carbon sequestration (43.59 million yuan), oxygen release (28.07 million yuan), air purification (4.73 million yuan), and wind and sand control (0.43 million yuan).

The value of cultural service products in the Taishan Scenic and Historic Spot was 2.19 billion yuan. Cultural services include two major indicators: tourism and recreation and scientific research and education. In 2023, the Taishan Scenic and Historic Spot received approximately 10.09 million tourists. The input of scientific research was 1110.68 yuan/ha. After accounting, the value of the tourism and recreation of the forest ecosystem at the Taishan Scenic and Historic Spot was 2175.75 million yuan, and the value of scientific research and education was 10.01 million yuan.

## 4 Discussion

### 4.1 Status analysis of forest GEP in the Taishan Scenic and Historic Spot in 2023

Among the indicators of regulating services, climate regulation had the greatest value, accounting for 80.28% of regulating services. This value is owing to the high altitude of the Taishan Scenic and Historic Spot, whose main peak, Yuhuangding, is the highest peak in Shandong at 1,545 m above sea level, with a height of 1,378 m relative to the foot of the mountain. The temperature difference inside and outside the ecosystem is considerable, and the service function of forest ecosystem climate regulation plays an obvious role. This indicator was followed by forest conservation, accounting for 5.25% of regulating services. This high value is because Taishan Mountain is located within a national nature reserve. In recent years, ecological protection and restoration projects involving mountains, water, forests, fields, lakes, and grasses and the protection of rare, endangered, and endemic species have been implemented in the Taishan region[49]. A good environment for biodiversity growth has been created through the management of damaged mountains, the innovative construction of a wild bird protection platform and the optimization of the living environment for wild birds. The conservation function of the ecosystem is energetic. The third- and fourth-ranked indicators of value were water conservation and floodwater storage. The Taishan Scenic and Historic Spot has a temperate monsoon climate, with an average precipitation of 758 mm[50] and concentrated summer rainfall, and the ecosystem flood storage and water conservation services regulate these systems, facilitating timely flood interception and even flood avoidance. These two indicators had large values, reflecting the strong regulatory capacity of water resources by the Taishan Scenic and Historic Spot. The values of windbreak and air purification were the smallest, accounting for less than 1%, which suggests the need to establish sand control and purification plantings to improve air quality.

**Fig 3:**
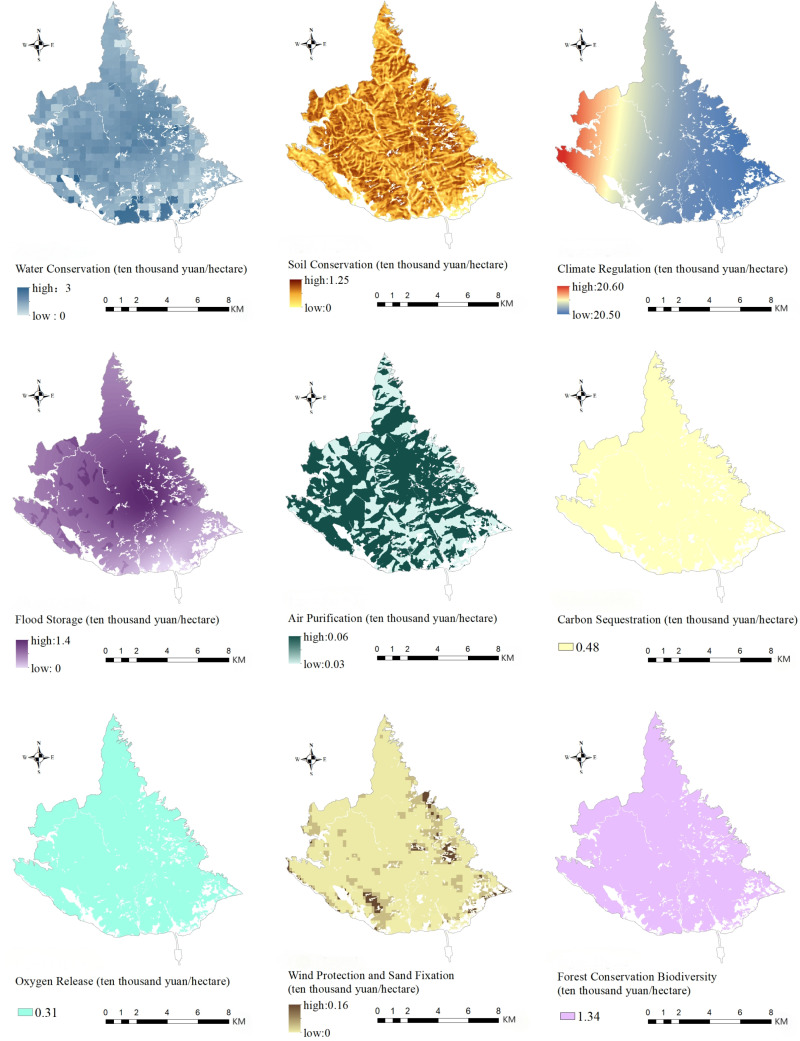
Spatial distribution of regulating services.

**Fig. 4:**
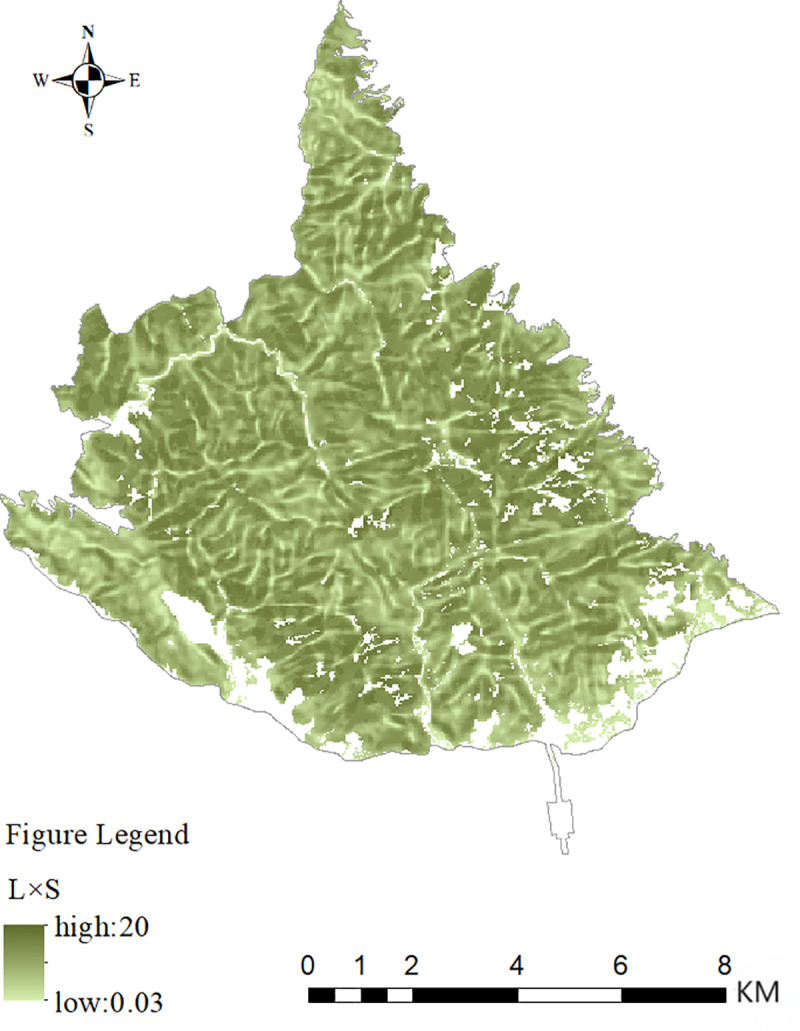
Products of the slope factor and slope length factor display chart.

The spatial distributions of various indicators of forest ecosystem-regulating services at the Taishan Scenic and Historic Spot are shown in Fig 3. The water conservation value tended to be high in the center and low in the surrounding area. This indicator is determined mainly by rainfall. Within the Taishan Scenic and Historic Spot in 2023, the precipitation in the center was significantly greater than that in the surrounding areas. The distribution of the soil conservation value was similar to the distribution map of the slope factor and slope length factor in the area, which is shown in Fig 4. Topography is the main factor influencing soil erosion[51], confirming the validity of the results of accounting for soil conservation values. The climate regulation value tended to be high in the west and low in the east. This difference is due mainly to the number of days with temperatures above 26 degrees Celsius. Interpolation with data from the temperature monitoring sites in Tai’an city yielded results that were slightly higher on the east side, but the differences between east and west were insignificant. The flood storage value generally tended to be high in the middle and low in the surrounding areas. This divergence is due to the difference between the storm water volume and the storm water runoff volume. The amount of rainfall in the Taishan Scenic and Historic Spot in 2023 was more concentrated in the middle of the area. The amount of air purification had an irregular distribution among forest types, which manifested, in order from greatest to smallest, as coniferous forest>  broad-leaved forest>  mixed coniferous and broad-leaved forest>  sparse forest. These amounts are determined mainly by the ecosystem type. Some studies have shown that the air purification capacity of different forest types differs and that the adsorption and purification capacity of coniferous forests is greater than that of broad-leaved forests[52]. These findings confirm, to some extent, the accuracy of the accounting results of the climate regulation indicator. There was no obvious pattern in the value distribution of the amount of value of wind protection and sand fixation. This distribution is influenced mainly by potential relative evaporation and vegetation cover factors. The values of the carbon sequestration, oxygen release and forest conservation indicators were related only to the area, so there was no difference in spatial distribution.

Among the cultural services, the tourism and recreation value was the highest, accounting for 99.54%, making it the main component of cultural service value; this value is owing to the rich tourism and cultural resources of Taishan[53]. The Taishan Scenic and Historic Spot has a double heritage of world culture and nature, and Taishan culture symbolizes “national peace”, with its spiritual connotation of civilization in China and abroad[30]. Mount Tai is located in the mountainous area of central Shandong and has convenient transportation, and buses and cableways are also available in the scenic area. With its superior location and transportation facilities, the Taishan Scenic and Historic Spot and the intensive cultural activities, temple fairs, youth studies, and other rich activities in the area have driven cultural tourism consumption. Taishan has a deep heritage and needs to continue to develop its cultural creativity and characteristics, as its potential for cultural value has yet to be tapped.

### 4.2 Characterization of the composition of forest GEP

**Fig. 5:**
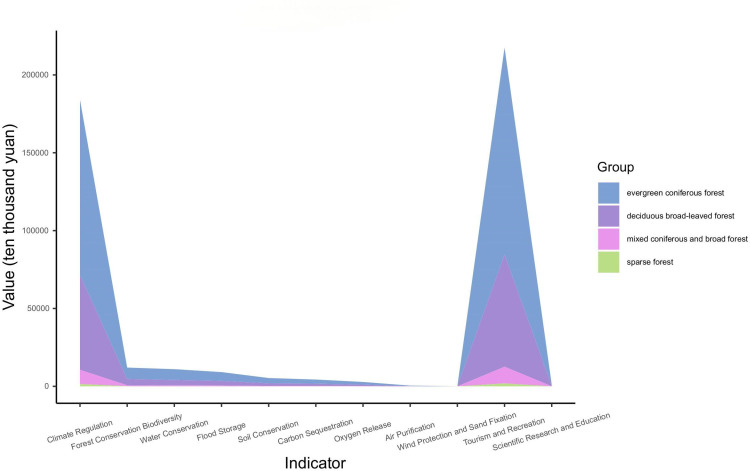
Characterization of the composition of the GEP.

The ArcGIS field calculator was used in the secondary classification of forest ecosystems to assign values to the forest ecosystem type codes for GEP accounting and to classify the forests in the Taishan Scenic and Historic Spot into broad-leaved forests, coniferous forests, mixed coniferous and broad-leaved forests, and sparse forests. In the accounting for the different service function types from the perspective of secondary classification of forest ecosystems, the GEP of evergreen coniferous forests was 274136 ten thousand yuan, accounting for 61.21%; the GEP of deciduous broad-leaved forests was 147784 ten thousand yuan, accounting for 33.00%; the GEP of mixed coniferous–broad-leaved forest was 22176 ten thousand yuan, accounting for 4.95%; and the GEP of sparse forests was 3738 ten thousand yuan, accounting for 0.83%. The analyzed results are shown in Fig 5.

The different types of forests in the Taishan Scenic and Historic Spot are ranked in order of size as follows: evergreen coniferous forest (5507 ha)>  deciduous broad-leaved forest (2983 ha)>  mixed coniferous and broad-leaved forest (445 ha)>  sparse forest (78 ha). Some studies have shown that the ability of ecosystems to provide services depends on the ecosystem area[54]. The results of the study revealed that the size rankings of the GEP values were the same as those of the ecosystem area, which somewhat confirmed the accuracy of these accounting results.

### 4.3 Analysis of changes in forest GEP

From 2022 to 2023, the forest GEP of the Taishan Scenic and Historic Spot increased from 2.13 billion yuan to 4.48 billion yuan. Among these increases, regulating services increased from 1.04 billion yuan to 2.29 billion yuan, an increase of 120.68%. Cultural services increased from 1.09 billion to 2.19 billion, an increase of 101.02%.

**Fig. 6:**
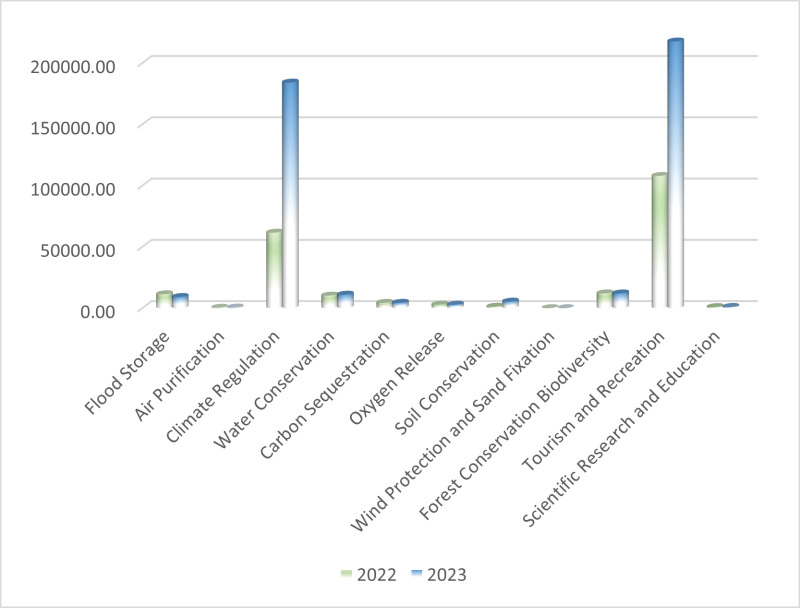
Analysis of changes in forest GEP in the Taishan Scenic and Historic Spot.

As shown in Fig 6, from 2022 to 2023, the indicators that achieved positive growth were air purification, climate regulation, water conservation, soil conservation, wind protection and sand fixation, tourism and recreation, and scientific research and education, with increases of 33%, 199%, 8%, 359%, 499%, 102%, and 17%, respectively. In contrast, flood storage demonstrated negative growth, with a growth rate of -19%. The indicators for carbon sequestration, oxygen release and forest conservation demonstrated rates of change of less than 0.01%, which is considered an accounting inaccuracy.

Compared with that in 2022, the largest growth rate, for wind protection and sand fixation, was due mainly to higher wind speeds in 2023. Vegetation helps make soil more wind resistant, reducing wind erosion of the soil and wind and sand hazards[55]. Soil conservation was the next-greatest contributor to the increase in value, mainly because of the increase in erosive rainfall compared with that in the previous year. Forest ecosystems have had ample opportunities to reduce the erosive capacity of rainfall and prevent soil erosion. The third-largest growth rate was for climate regulation, which is due to the higher temperatures in 2023. The vegetation absorbed more energy through transpiration and thus regulated the temperature more effectively. The rate of change in the value of tourism and recreation also exceeded 100%. This occurred because, in 2023, the effects of China’s new coronavirus epidemic receded, and the economy began to recover, thus contributing significantly to the tourism boom[56]. The Taishan Scenic and Historic Spot experienced a significant increase in foot traffic, and the value of cultural services doubled as a result.

## 5 Conclusions

Just as GDP measures the economy and development of a region, we also need GEP to measure the contribution of nature to human well-being. To address the challenges of the existing accounting system not being targeted to account for the forest GEP value in nature reserves, this work proposes additions on the basis of the Technical Guidelines for Accounting for the Gross Value of Ecological Products in Shandong Province. An accounting system for forest GEP that is applicable to nature reserves is established. This work applies a system to establish a “Taishan Sample” for accounting for ecological products. The accounting results provide data-based support for realizing the value of ecological products in the Taishan Scenic and Historic Spot, as well as a reference for understanding the mechanism of sustainable ecosystem development. The specific conclusions are as follows:

1. Accounting for the GEP of forests in nature reserves can provide decision makers with clear data on the monetary value of ecosystem services. This study adds indicators of scientific research and education and of forest conservation biodiversity value to the GEP accounting system, which provides a reference for forest ecosystem valuation in other nature reserves. This study contributes to the realization of the value of GEP, for example, by providing evidence for ecological compensation, which contributes to the achievement of sustainable development goals for society as a whole.
2. This study accounts for the forest GEP of the Taishan Scenic and Historic Spot in 2022. This work involved both a trial of the accounting system presented herein and an assessment of the Taishan Scenic and Historic Spot. This work lays the foundation for quantifying the value of nature reserves.
3. On the basis of the accounting results, the spatial distribution of forest GEP in the Taishan Scenic and Historic Spot in 2023 is presented, and the current status of its ecosystem services is analyzed. This study also summarizes the trend of forest GEP in the Taishan Scenic and Historic Spot from 2022 to –2023 and analyses the reasons for the significant value changes in the indicators. This analysis provides a direction for promoting the transformation of ecological value to economic value in the Taishan Scenic and Historic Spot.
4. The forest ecosystems of the Taishan Scenic and Historic Spot were classified at the second level. An analysis of the value distributions of coniferous forests, broad-leaved forests, mixed coniferous and broad-leaved forests and sparse forests revealed that the forest GEP depended considerably on the area of the ecosystem. A new research perspective is provided for the further classification of forest ecosystem types.
5. Research on the GEP of forests is in a stage of continuous development and improvement, through which the ecological service functions of forests can be fully interpreted. However, there may be additional factors in the GEP accounting system that are not included in the present work. In addition to the GEP accounting metrics presented here, ecosystems have other service ecological products, such as rainfall pattern regulating services, solid waste remediation services, and pollination services. To provide a more complete picture of global environmental benefits, it is necessary to continue studying ecosystems in depth and to clarify how ecosystems contribute to humanity. In the future, we will conduct further research on ecosystem values.

## Supporting information

S1 Text Forest GEP accounting model for nature reserves.
Specific accounting methods for the 11 GEP indicators considered in this study, including the value calculation process and required parameters.
(DOCX)
