# Supplementary material for: Accounting for the gross ecosystem product (GEP) of forests in nature reserves—taking the Taishan Scenic and Historic Spot as an example
Source: PLoS One. 2025 Mar 25;20(3):e0320075. doi: 10.1371/journal.pone.0320075 (PMC11936196; doi:10.1371/journal.pone.0320075)
Supplement: S1 Text — Specific accounting methods for the 11 GEP indicators considered in this study, including the value calculation process and required parameters. (DOCX) [file pone.0320075.s001.docx]

**Forest GEP Accounting Model for Nature Reserves**

**1 GEP Accounting Model**

$PVRS=V_{wr}+V_{sr}+V_{tt}+V_{fm}+V_{ap}+V_{Cf}+V_{op}+V_{sf}+V_{cb}$…………（1）

$PVCS=V_{r}+V_{SC}$……………………………………（2）

$GEP=PVRS+PVCS$……………………………………（3）

The formula:

***PVRS***——Product Value of Regulating Services(yuan/a)；

***V_wr_***——water conservation value of forest ecosystem(yuan/a)；

***V_sr_****——*soil conservation value of forest ecosystem(yuan/a);

***V_tt_***——climate regulation value of forest ecosystem(yuan/a);

***V_fm_***——flood storage value of forest ecosystem(yuan/a);

***V_ap_***——air purification value of forest ecosystem(yuan/a);

***V_Cf_***——carbon sequestration value of forest ecosystem(yuan/a);

***V_op_***——oxygen release value of forest ecosystem(yuan/a)；

***V_sf_***——wind protection and sand fixation value of forest ecosystem(yuan/a)；

**𝑉_cb_***——*forest conservation biodiversity value of forest ecosystem(yuan/a)；

***PVCS****——*Product value of Cultural Services (yuan/a)；

***V_r_*** *——*tourism and recreation value of forest ecosystem(yuan/a)；

***V_SC_****——*scientific research and education value of forest ecosystem(yuan/a)；

***GEP****——*Gross Ecosystem Product of forest ecosystems in nature reserves(yuan/a)；

**2 Accounting Models for the Value of Regulating Service Products**

**2.1 Water Conservation**

Physical quantity：Water Balance Model。

$Q_{wr}=\sum_{i=1}^{n} A_{i}\times\left( P_{i}-R_{i}-{ET}_{i} \right)\times{10}^{3}$ …………………… (4)

The formula:

***Q_wr_***——amount of water conservation in forest ecosystem(m^3^/a);

***A_i_***——area of forest ecosystem of category ***i*** (km^2^)；

***P_i_***——amount of rainfall(mm/a);

***R_i_***——amount of surface runoff(mm/a);

***ET_i_*** ——the amount of evapotranspiration(mm/a)；

***i***——type of forest ecosystem, ***i*** =1,2,3,...,***n***；

***n***——number of types of forest ecosystem.

Value quantity：Alternative Costing Method。

$V_{wr}=Q_{wr}\times\left( C_{we}+P_{we}\times D_{r} \right)$…………………………………(5)

The formula:

***V_wr_***——water conservation value of forest ecosystem(yuan/a)；

***Q_wr_***——amount of water conservation of forest ecosystem(m^3^/a);

***P_we_***——engineering cost of reservoir unit capacity(yuan/m^3^)；

***C_we_***——annual operating cost of reservoir unit capacity（yuan/（m^3^• a））；

***D_r_***——annual depreciation rate of the reservoir.

**2.2 Soil Conservation**

Physical quantity：Modification of the Generalized Soil Loss Equation。

$Q_{sr}=\sum_{i=1}^{n} \left[ R_{i}\times K_{i}\times L_{i}\times S_{i}\times\left( 1-C_{i} \right)\times A_{i}\times{10}^{2} \right]$…………………… (6)

The formula:

***Q_sr_***——amount of soil conservation in forest ecosystem(t/a);

***A_i_—***—area of accounting unit ***i***(km^2^);

***i***——accounting unit, ***i = 1,2,3, ...,n***；

***n***——the number of accounting units；

***R_i_***——rainfall erosivity factor for accounting unit ***i*** (MJ • mm/ (hm^2^ • h • a))；

***K_i_***——soil erodibility factor for accounting unit ***i*** (t • hm^2^ • h/(hm^2^ • MJ •mm))；

***L_i_***——slope length factor of accounting unit ***i***(dimensionless)；

***S_i_***——slope factor of accounting unit ***i*** (dimensionless)；

***C_i_***——vegetation cover factor of accounting unit ***i*** （dimensionless）;

Value quantity：Alternative Costing Method。

$V_{sr}=V_{sd}+V_{dpd}$ *………*……………………*……………*(7)

$V_{sd}=\lambda\times\left( \frac{Q_{sr}}{\rho} \right)\times c$……………………………………… (8)

$V_{dpd}=\sum_{i=1}^{n} Q_{sr}\times c_{i}\times p_{i}$ ………………………(9)

The formula:

***V_sr_——***value of soil conservation in forest ecosystem（yuan/a）;

***V_sd_——***value of reduction of sedimentation（yuan/a）；

***V_dpd_——***value of reduction of surface pollution（yuan/a）;

***λ——***sediment deposition coefficient (dimensionless);

***Q_sr_——***the amount of soil conservation in forest ecosystem（t/a）；

***ρ——***soil capacity weight（t/m^3^）；

***c——***cost of reservoir unit dredging works（yuan/m^3^）；

***c_i_——***pure content of pollutants of class ***i*** (e.g. nitrogen, phosphorus) in soil（％）；

***P_i_——***unit treatment cost of pollutants in category ***i***（yuan/t）;

***i——***category of pollutants in the soil，***i***=1,2,3,. ..，**n**；

***n——***number of pollutant categories in soil.

**2.3 Climate Regulation**

Physical quantity：Vaporization Model。

$E_{pt}=\sum_{i}^{n} {EPP}_{i}\times S_{i}\times D\times{10}^{6}/\left( 3600\times r \right)$………………………(10)

The formula:

***E_pt_***——energy consumed by evapotranspiration in forest ecosystem(kW • h/a);

***EPP_i_***——heat consumed by evapotranspiration per unit area of forest ecosystem of category ***i*** (kJ/(m^2^ • d))；

S***_i_***——area of forest ecosystems of category ***i*** (km^2^);

r——energy efficiency ratio of air conditioning(dimensionless)；

***D***——number of days open to air conditioning for cooling(d/a);

***i***——forest ecosystem type *i*=1,2,3,...，n;

***n***——number of forest ecosystem types.

Value quantity：Alternative Costing Method。

$V_{tt}=E_{pt}\times P_{e}$…………………………………（11）

The formula:

***V_tt_***——value of climate regulation of forest ecosystem（yuan/a）;

***E_pt_***——Total energy consumed by forest ecosystem to regulate temperature and humidity （kW • h/a）；

***P_e_***——local living consumption electricity price（yuan/kW • h）。

**2.4 Flood Storage**

Physical quantity：Water Balance Model。

$C_{vfm}=\sum_{i=1}^{n} \left( P_{i}-R_{fi} \right)\times A_{i}\times{10}^{3}$ ………………………（12）

The formula:

***C_vfm_***——the amount of flood storage in forest ecosystem(m^3^/a);

***P_i_***——the amount of storm rainfall (mm/a);

***R_fi_***——storm water runoff volume of forest ecosystem of category ***i***

(mm/a);

***Ai***——area of forest ecosystem of type ***i*** (km^2^);

***i***——type of forest ecosystem，***i*** =1,2,3,...,n；

***n***——number of forest ecosystem types.

Value quantity：Alternative Costing Method。

$V_{fm}=C_{vfm}\times\left( C_{we}+P_{we}\times D_{r} \right)$………………………（13）

The formula:

***V_fm_***——value of flood storage in forest ecosystem（yuan/a）;

***C_vfm_***——the amount of flood water storage in the forest ecosystem（m^3^/a）；

***P_we_***——engineering cost of reservoir unit capacity（yuan/m^3^）

***C_we_***——annual operating cost of reservoir unit capacity(yuan/(m^3^• a));

***D_r_***——annual depreciation rate of the reservoir.

**2.5 Air Purification**

Physical quantity：Pollutant Purification Model

$Q_{ap}=\sum_{i=1}^{n} \sum_{j=1}^{m} Q_{ij}\times A_{j}$…………………………………(14)

The formula:

***Q_ap_***——the amount of air purified by forest ecosystem(t/a);

***Q_ij_***——the amount of purification of air pollutants of category ***j*** ecosystems per unit area of category ***i*** （t/km2 ·a）;

***i***——category of atmospheric pollutants, ***i=1,2,3,...n***；

***n——***number of categories of atmospheric pollutants;

***j——***type of ecosystem，***j=1，2，…，m***；

***m——***number of ecosystem types；

***A_𝑗_ ——***area of ecosystem type ***j*** （km^2^）。

Value quantity：Alternative Costing Method。

$V_{ap}=\sum_{i=1}^{n} Q_{i}\times c_{i}$ ………………………………（15）

The formula:

***V_ap_***——the value of air purification of forest ecosystem（yuan/a）;

***Q_i_***——purification volume of air pollutants of category ***i***（t/a）;

***i***——category of air pollutants, ***i*** =1,2,3,... ,*n；*

***n***——the number of categories of atmospheric pollutants；

***c_i_***——unit treatment cost of air pollutants of category ***i***（yuan/t）。

**2.6 Carbon Sequestration**

Physical quantity：Carbon Sequestration Rate Model。

$Q_{{tCO}_{2}}=\frac{M_{{CO}_{2}}}{M_{C}}\times\left( FVCSR+FSCSR \right)\times SF$*………………………* (16)

The formula:

***Q_t_co_2_***——the amount of carbon sequestered in forest ecosystem(t • CO_2_/a)；

***M_CO2_/M_c_* =44/12**——coefficient of conversion of C to CO_2_；

***FVCSR***——the rate of carbon sequestration by vegetation in forest ecosystem(t • C/ (hm^2^ • a));

***FSCSR***——carbon sequestration rate of forest ecosystem soil(t • C/ (hm^2^ • a))；

***SF***——area of forest ecosystem(hm^2^)。

Value quantity：Alternative Costing Method。

$V_{Cf}=Q_{{tCO}_{2}}\times C_{{CO}_{2}}$………………………………… (17)

The formula:

***V_Cf_***——value of carbon sequestered by forest ecosystem(yuan/a);

***Q_t_co_2_***——the amount of carbon sequestered in forest ecosystem(t • CO_2_/a)；

***C_C02_***——carbon dioxide price(yuan/t • CO_2_).

**2.7 Oxygen Release**

Physical quantity：Oxygen Release Mechanism Model for Carbon Sequestration

$Q_{top}=M_{O_{2}}/M_{{CO}_{2}}\times Q_{{tCO}_{2}}$……………………………… (18)

The formula:

𝑄_𝑡𝑜𝑝_*——*the amount of oxygen released from the forest ecosystem（tO_2_/a）；

M_O2_/𝑀_𝐶𝑂2_ =32/44*——*the coefficient of conversion of CO_2_ to O_2_；

𝑄_𝑡𝐶𝑂2_*——*the amount of carbon sequestered by forest ecosystem（tCO_2_/a）.

Value quantity：Market Value Approach。

$V_{op}=Q_{top}\times C_{O}$……………………………………（19）

The formula:

*V_op_*——value of oxygen release from forest ecosystem（yuan/a）；

𝑄_𝑡𝑜𝑝_——amount of oxygen released from forest ecosystem（t·O_2_/a）；

𝐶_O_——price of industrial oxygen production（yuan/t·O_2_）。

**2.8** **Wind Protection and Sand Fixation**

Physical quantity：Modified Wind Erosion Modeling。

$Q_{sf}=\sum_{i=1}^{n} \left[ 0.1699\times\left( {WF}_{i}\times{EF}_{i}\times{SCF}_{i}\times K_{i}^{，} \right)^{1.3711}\times\left( 1-C_{i}^{1.3711} \right)\times A_{i} \right]$ …（20）

The formula:

***Q_sf_***——the amount of forest ecosystems to wind protection and sand fixation（t/a）；

***Ai***——rea of accounting unit ***i*** （km^2^）;

***i***——accounting unit ,*i*= 1,2,3,...,n；

***n***——the number of accounting units

***WF_i_***——climatic erosion factor of accounting unit ***i***（kg/m）;

***EF_i_***——soil erosion factor of accounting unit ***i*** (dimensionless)；

***SCF_i_***——soil crust factor for accounting unit ***i*** (dimensionless)；

***K_i_^’^***—— surface roughness factor for accounting unit ***i***(dimensionless)；

***C_i_***——vegetation cover factor (dimensionless) for accounting unit ***i***.

Value quantity：Recovery Cost Approach。

$V_{sf}=\frac{Q_{sf}}{\rho\cdot h}\times c$……………………………………（21）

The formula:

***V_sf_***——value of forest ecosystems for wind protection and sand fixation（yuan/a）；

***Q_sf_***——amount of forest ecosystem wind protection and sand fixation（t/a）;

***ρ***——soil bulk weight（t/m^3^）；

***h***——thickness of sand-covered soil sand (m) ;

***c***——unit cost of sand control project or unit cost of vegetation restoration（yuan/m^2^）_o_

**2.9 Forest Conservation Biodiversity**

Physical quantity：Statistical Surveys Act。

$G_{biop}=S$………………………………………（22）

The formula:

***G_𝑏𝑖𝑜𝑝_——***amount of species conservation function(m^2^)；

***S——***area of the nature reserve(m^2^).

Value quantity：Conservation Value Method。

$V_{bio}=G_{biop}\times S_{生}$…………………………………（23）

The formula:

**𝑉*_cb_——***value of forest conservation（yuan/a）；

**𝑆*——***value of species conservation per unit area（yuan/（hm^2^·a））

**3 Accounting Models for the Value of Cultural Service Products**

**3.1 Tourism and Recreation**

Physical quantity：Statistical Surveys Act。

$N=\sum_{i=1}^{n} N_{ti}$……………………………………（24）

The formula:

***N——***the total number of visitors to the natural scenic spots of forest ecosystem（人•次/a）；

***N_ti_——***the *i*th forest ecosystem natural scenic area of the number of tourists（人•次/a）;

***i——***type of ***forest*** ecosystem natural scenic area ***,i= 1,2,3, ... ,n***；

***n——***number of ***forest*** ecosystem natural scenic spots.

Value quantity：Travel Costs Act。

$V_{r}=N\times TC+\sum_{i=1}^{n} C_{i}$……………………………（25）

$TC=T\times W$……………………………………（26）

The formula:

***V_r_ ——***value of tourism and recreation services（yuan/a）；

***N——***total number of trips to the natural scenic area of the forest ecosystem（person • day /a）;

***TC——***average time cost of tourists traveling to natural scenic spots in forest ecosystem（yuan/（person • day））；

***T——***average time spent by tourists traveling the road and traveling in the natural scenic area of forest ecosystem（day/tinme）；

***W——***average wage of tourists（yuan/（person • day））；

***C_i_——***business income of the *ith* forest ecosystem scenic spot（yuan/a）;

***i——***forest ecosystem natural scenic spots，***i=*** *1,2,3, ... ,****n***；

***n——***the number of forest ecosystem natural scenic spots.

**3.2** **Scientific Research and Education**

Value quantity：Results-based Approach。

$V_{SC}=RD\times\left( {SG}_{gov}+{SG}_{edu} \right)\times A_{p}$………………………（27）

The formula:

***V_SC_——***value of scientific research and education in forest ecosystem（yuan/a）;

***RD­——***the total expenditure of funds actually incurred by the research region for the implementation of research and experimental development (R&D) activities in the base year（yuan/a）。

***SG_gov_——***the share of research and experimental development in R&D invested by government-owned research institutions;

***SG_edu_——***the share of research and experimental development in R&D in educational institutions;

***A_p_——***Proportion of area with scientific and educational value in the study area.
